# Correction for Brown-Elliott et al., "Utility of Sequencing the *erm*(41) Gene in Isolates of *Mycobacterium abscessus* subsp. *abscessus* with Low and Intermediate Clarithromycin MICs"

**DOI:** 10.1128/jcm.00887-25

**Published:** 2025-08-20

**Authors:** Barbara A. Brown-Elliott, Sruthi Vasireddy, Ravikiran Vasireddy, Elena Iakhiaeva, Susan T. Howard, Kevin Nash, Nicholas Parodi, Anita Strong, Martha Gee, Terry Smith, Richard J. Wallace

## AUTHOR CORRECTION

Volume 53, no. 4, p. 1211-1215, 2015, https://doi.org/10.1128/jcm.02950-14. Recent sequencing of the isolates designated sequevars 3 and 5 indicates that both isolates have the sequevar 2 sequence. Therefore, the entries for sequevars 3 and 5 in Tables 1 and 2 should be disregarded, and other references to sequevars 3 and 5 should be discounted or considered to refer to sequevar 2. All isolates that were designated as belonging to sequevar 2, 3, or 5 were macrolide susceptible.

Regrettably, the original records for this study are no longer available. The GenBank records for the two isolates originally classified as sequevars 3 and 5 (accession no. KP676135 and KP676137) were revised in the NCBI database on 18 July 2025 to indicate that these isolates have the sequevar 2 sequence.

